# Comparing Tolerability and Efficacy of Generic versus Brand Alendronate: A Randomized Clinical Study in Postmenopausal Women with a Recent Fracture

**DOI:** 10.1371/journal.pone.0078153

**Published:** 2013-10-21

**Authors:** Joop P. W. van den Bergh, Marian E. Bouts, Eveline van der Veer, Robert Y. van der Velde, Marcel J. W. Janssen, Piet P. Geusens, Bjorn Winkens, Nico J. J. Oldenhof, Tineke A. C. M. van Geel

**Affiliations:** 1 Department of Internal Medicine, VieCuri Medical Centre of Noord-Limburg, Venlo, The Netherlands; 2 Department of Internal Medicine, Maastricht University Medical Centre, Maastricht, The Netherlands; 3 Maastricht University, NUTRIM - School for Nutrition, Toxicology and Metabolism, Maastricht, The Netherlands; 4 Biomedical Research Centre, University Hasselt, Diepenbeek, Belgium; 5 Laboratory Medicine, University Medical Centre Groningen, Groningen, The Netherlands; 6 Laboratory of Clinical Chemistry and Haematology, VieCuri Medical Centre of Noord-Limburg, Venlo, The Netherlands; 7 Maastricht University, CAPHRI - School for Public Health and Primary Care, Maastricht, The Netherlands; 8 Department of Methodology and Statistics, Maastricht University, Maastricht, The Netherlands; 9 Hospital Pharmacy, VieCuri Medical Centre of Noord-Limburg, Venlo, The Netherlands; 10 Department of Family Medicine, Maastricht University, Maastricht, The Netherlands; Georgia Regents University, United States of America

## Abstract

**Introduction:**

An increasing number of generic alendronate formulations have become available. Although expected to have the same tolerability and efficacy, head-to head comparison of generic and brand alendronate was never performed. Therefore, we compared the tolerability and efficacy of generic and brand alendronate.

**Methods:**

In a randomized double-blinded single centre cross-over study in 37 postmenopausal women (mean age 65.4±6.4 years) with osteoporosis were treated with generic and branded alendronate during 24 (2x12) weeks. Tolerance was evaluated by the Gastro intestinal Symptom Rating Scale (GSRS) and self-reported side effects. Efficacy was assessed by serum bone turnover markers, carboxy terminal telopeptide (CTX) and procollagen type I N-terminal propeptide (PINP). No wash out period was allowed (ethical reasons). Because of possible carry over effect only data of the first 12 weeks were analyzed using linear mixed models.

**Results:**

There were no significant differences in overall tolerance (GSRS) between treatment groups. However, for subscale abdominal pain, patients using generic had a significantly higher mean GSRS score at week 4 (estimated mean difference (B): 0.40; 95%CI: 0.05 to 0.74, p = 0.024). The level of bone turnover markers significantly decreased over 12 weeks of follow-up for generic and branded alendronate (p < 0.001). Mean level of CTX was significantly lower with branded at week 4 (B: 121.3; 95%CI: 52.0 to 190.5), but not at week 12 (B: 53.6; 95%CI:-3.7 to 110.9). No significant differences were found for PINP at week 4 or 12.

**Conclusions:**

Bone turnover markers were significantly reduced with branded and generic alendronate. With branded, CTX was significantly lower at 4 weeks. Generic caused significantly higher abdominal pain scores in the first 4 weeks of treatment. Therefore, generic alendronate may not have the same tolerability and efficacy as branded alendronate in the first weeks after starting treatment in patients with a recent fracture.

**Trial Registration:**

Dutch Trial Register NTR number 1867 http://www.trialregister.nl/trialreg/admin/rctview.asp?TC=1867

## Introduction

Due to the ageing population and consequently the increasing incidence of fractures and its related morbidity and mortality, osteoporosis has extensive clinical and economic consequences. About 50% of the woman and 20% of the men older than 50 years will have a fragility fracture during their remaining lifetime. In the Netherlands, the number of fragility fractures is estimated at more than 80.000 per year [1]. 

Every fragility fracture signals increased risk of future fractures as well as risk of premature mortality [2], and therefore, at present routine assessment of patients after a recent fracture at a Fracture Liaison Service is implemented in many hospital in different countries[3–9]. As a consequence of the Fracture Liaison Service assessment appropriate pharmacological intervention according to local guidelines is started in those patients shortly after they sustained a fracture. In the Dutch guidelines, anti-resorptive treatment with the bisphosphonates alendronate or risedronate is considered as first choices treatment [1]. These bisphosphonates have been shown to significantly reduce the risk of vertebral, non-vertebral and hip fractures in randomized clinical trials of postmenopausal women with osteoporosis[10,11]. On the one hand, bisphosphonates inhibit bone resorption and decrease the levels of bone turnover makers [12]. On the other hand, bone turnover is elevated up to 12 months after fracture with a reported significant increase of formation and bone resorption markers 4 months after fracture [13], a time-point when most patients are or already have been assessed at the Fracture Liaison Serviceand treatment is initiated. 

During recent years an increasing number of generic alendronate and more recently risedronate formulations were introduced in several countries (also in the Netherlands). The generic formulation is expected to have the same clinical efficacy as the branded formulation based on bioequivalence studies in which the pharmacokinetic profile (urinary excretion) of the branded formulation was compared to the reference generic product in a group of healthy volunteers [14]. Insurances and Health Care Providers prefer physicians to prescribe mainly generic alendronate instead of branded original bisphosphonates, based on lower costs. Although it is expected to have the same clinical efficacy, clinical information on BMD and fracture reduction, adverse effects and compliance with new generic alendronates is sparse. Additionally the effect of branded versus generic alendronate has not been studied in patients treated shortly after they sustained a fracture. Therefore, the aim of this study is to evaluate the potential differences in efficacy and tolerability between branded versus generic alendronate in postmenopausal women with a recent fracture.

## Subjects and Methods

The protocol for this trial and supporting CONSORT checklist are available as supporting information (Checklist S1 and Protocol S1). A randomized single centre cross-over study in postmenopausal women with a recent fracture was conducted over a period of 24 (two times 12) weeks. After signing informed consent, patients enrolled into the study between December 2009 and February 2011. Women were randomized to start with generic (Accord, RVG 100474) or branded alendronate (Fosamax®, manufactured by MSD), in a once weekly oral dose of 70 milligram (allocation ratio 1:1). The generic alendronate (Accord, RVG 100474) used in this study was preferred and the only reimbursed formulation of alendronate by the health insurance in the region of the VieCuri Hospital. Randomization to generic versus brand alendronate at the start of the study was performed by the site pharmacist by selecting previously prepared closed envelopes. Patients received first oral generic alendronate for 12 weeks, after cross-over followed by branded alendronate for 12 weeks or vice versa. The medication was taken out of the original package and provided to the women in unlabelled containers. They received the medical leaflet of both medications at the baseline visit. All women and investigators were blinded for medication. During the entire study period all women received calcium (1000mg/day) and vitamin D (800 IU/day). 

Study visits were planned at baseline, week 4, 12, 16 and 24. Due to ethical reasons, there was no wash-out period in the study design. At each visit, blood samples were collected, questionnaires were completed and study medication was provided and return medication was collected and counted by the hospital pharmacist. The number of tablets provided at each visit varied and exceeded the number needed for the study period until the next study visit. Therefore, women were instructed to return the residual study medication at each visit for pill counts. A study nurse evaluated possible side effects and adverse events. Worsening of a pre-existing medical condition (increase of medical condition in severity, frequency or duration), occurrence of a fracture and clinically relevant laboratory abnormalities or adjustments in prior therapy were considered adverse events. 

Tolerance, as measured by Gastro Intestinal Symptom Rating Scale (GSRS)-scores, self-reported side effects and efficacy, as measured by bone turnover markers carboxy terminal telopeptide (CTX) and procollagen type I N-terminal propeptide was also assessed.

### Participants

All women were recruited from the Fracture Liaison Service of VieCuri Medical Centre Noord-Limburg after regular assessment of clinical risk factors, dual X-ray absorptiometry (DXA) and laboratory evaluation. Details of the Fracture Liaison Service assessment are previously described [5]. If treatment was indicated according to the Dutch Institute of Health Care Improvement guideline on osteoporosis [1], patients were asked to participate in this study. Inclusion criteria of the study were: postmenopausal women (defined as no vaginal bleeding or spotting for at least 12 months), ambulatory and older than 50 years of age, with a non-vertebral fracture and diagnosed with osteoporosis (DXA T-score ≤ -2,5 either at the lumbar spine, femoral neck or total hip), and who were able to understand study procedures, drugs and dose instructions. Time of inclusion must be longer than 3 months post-fracture and 6 months post hip fracture, and women had to be adequately recovered from their fracture. Women were excluded if they were treated with anti-osteoporosis medication (bisphosphonates, testosterone, hormone replacement therapy, selective estrogen receptor modulators or calcitonin) within 12 months before inclusion, known intolerance for bisphosphonates, disorders of esophageal motility or contra-indications of oral bisphosphonates, known secondary osteoporosis (primary hyperparathyroidism, renal disease, untreated primary hyper- of hypothyroidism) or malignancy within the last 5 years and alcohol use of more than 4 units per day. 

### Assessments

Gastrointestinal symptoms were evaluated at all visits using the Gastrointestinal Symptom Rating Scale (GSRS) questionnaire[15]. This questionnaire with 15 items is divided into five subscales (abdominal pain, diarrhoea, constipation, indigestion and reflux). For each item a score from 0 (no complaints) to 6 (serious complaints) can be given. Per subscale the scores are calculated as the mean of the items completed within the subscale and the overall score is the mean of the 5 subscale means[15]. Higher scores indicate greater severity of symptoms. The GSRS has good reliability and construct validity. Besides the GSRS, self-reported side effects were monitored at each visit. 

Medication use and adherence were evaluated by pill counts, the Self-Efficacy for Appropriate Medication use (SEAMS) Questionnaire and the Brief Medication Questionnaire (BMQ). The SEAMS questionnaire is a reliable and valid tool that may provide a valuable assessment of medication self-efficacy in chronic disease management [16]. The BMQ is also validated and can be used to identify and diagnose adherence problems [17]. The results of the BMQ are classified into three categories based on a scoring procedure for BMQ screens. The regimen screen measures adherence behaviour, the belief screen measures the patient’s feelings about the efficacy and unwanted side effects or concerns of the given medication and the recall screen measures potential problems remembering all doses [17]. Both SEAMS as BMQ were evaluated at week 4, 12, 16 and 24. Medication adherence was defined as having taken at least 80% of the prescribed tablets in both treatment periods of twelve weeks and was evaluated by pill count. At baseline and after 24 weeks a general physical examination was performed by a physician. 

### Biochemical assessments

Differences in efficacy of generic and branded bisphosphonate were evaluated by use of bone turnover markers. One of the assessed bone turnover markers is carboxy terminal telopeptide (CTX), which is a collagen resorption marker. A marker of bone formation was also assessed, namely procollagen type I N-terminal propeptide (PINP). Both markers are measured in blood serum samples. 

Blood samples were collected at all visits. Fasting serum samples were collected. PINP was measured by radioimmunoassay (RIA; Orion Diagnostica, Espoo, Finland; inter-assay coefficient of variation (IE-CV) 9.0%). sCTX was measured by electrochemiluminescence immunoassay (ECLIA; Elecsys 2010 Roche Mannheim, Germany; IE-CV 10.8%). Basic laboratory tests were performed at baseline, week 12 and 24 and included serum calcium, phosphate, albumin, 25 (OH) vitamin D3, parathyroid hormone (PTH), sodium, potassium, creatinine and hemoglobin. 

### Statistical methods

SPSS software (version 21.0) was used for the statistical analyses (SPSS Inc. Chicago, IL, USA). Baseline characteristics were compared between the groups using Chi-square test or Fisher’s exact test, where appropriate, for categorical variables, and independent-samples t-test or Mann-Whitney U-tests, if data were not normally distributed, for numerical variables. 

Bone turnover markers are expressed in absolute values and in Z-scores. Z-scores of bone turnover markers were used to correct for the normal influence that age and gender have on bone turnover. Z-scores, the number of standard deviations (SD) from the normal mean for age and gender, were calculated using matched 10-year-cohorts of a Dutch reference group (350 women), checked for serum 25OH vitamin D levels > 50 nmol/liter as well as for lumbar spine and hip BMD T-score > -2.5 after 50 years of age [18].

Due to ethical reasons it was not allowed to include a wash out period and as could be expected a carry-over effect was found for the bone markers, not for the adherence, tolerance and self-reported side effects outcomes. Based on these findings, only data before the cross-over period (baseline, week 4 and 12) were taken into account for the analyses. 

For tolerance (as measured by GSRS), treatment effect (generic versus branded alendronate) was analyzed using a random intercept model with (1) time, (2) group and (3) time*group as fixed factors to account for the correlation between repeated measurements within the same subject. The same model was used for number of self-reported side effects. For bone turnover markers (CTX and PINP) random intercept models with (1) time, (2) group, (3) time*group and with or without including (4) tolerance as fixed factors were performed. Tolerance was included to study the additional effect of treatment on the bone turnover markers. Adherence (by mean pill count, SEAMS, and BMQ) were analysed by T-test and Chi-square test (for week 4 and 12). A p-value ≤ 0.05 was considered statistically significant. Data were analyzed according to the intention-to-treat principle.

Sample size is calculated (N = (Zalpha + Zbeta)2 * SD2 / 2d2; paired observations, alpha one sided)[19] based on α = 0.05 (95% confidence interval), β = 0.1 (power 90%), with a standard deviation of sCTX measurements of 0.5 (SD) and d as the mean of the difference between both treatment groups. Considering a serum CTX Z-score difference of < 0.5 SD between both groups as not clinically relevant, a sample size of 36 patients is needed. 

### Ethics Statement

This study was approved by the medical ethical committee of Maastricht University Medical Centre, The Netherlands and registered in the Dutch trial register; NTR number 1867, http://www.trialregister.nl/trialreg/admin/rctview.asp?TC=1867.

All patients who were willing and able to participate signed an informed consent form. After signing informed consent, patients enrolled into the study between December 2009 and February 2011.

## Results

**Figure 1:**
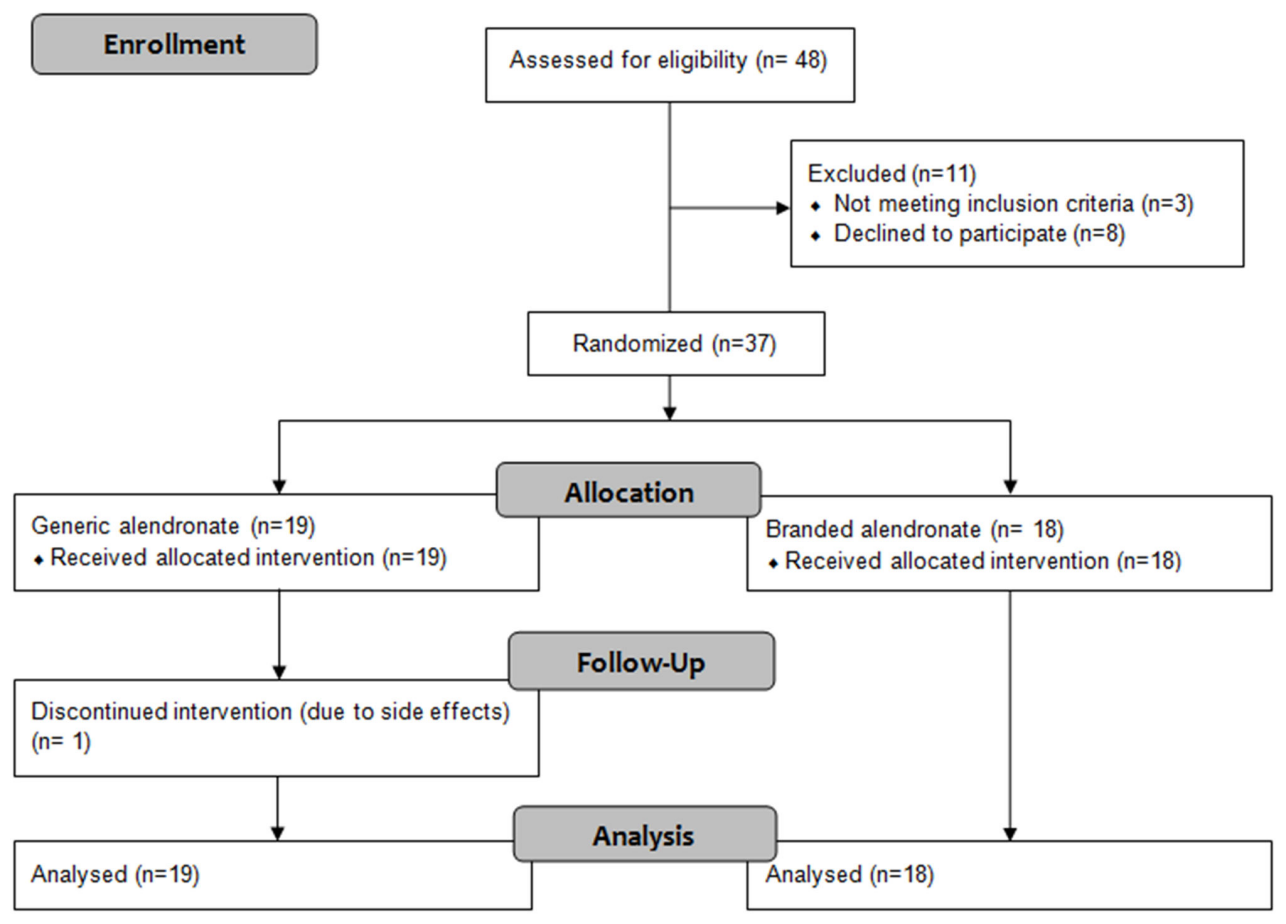
Flow chart for 12 weeks follow-up.

A total of 37 postmenopausal women with osteoporosis were included in the study. After randomisation, 19 patients started with generic and 18 with branded alendronate (Figure 1). Baseline characteristics are presented in Table 1. At baseline, only vitamin 25(OH)D3 levels were significantly different between the two treatment groups (p = 0.005).

**Table 1:** Baseline characteristics of patients (mean ± SD for continuous variables and number (%) for categorical variables) specified for treatment (generic or branded alendronate).

**Characteristic**	**Generic alendronate (n=19)**	**Branded alendronate (n=18)**	**P value**
Mean age (years)^a^	66.7 ± 6.9	64.1 ± 5.8	0.226
History of fracture, n (%)^c^			0.138
hip, n (%)	1 (5.3)	1 (5.6)	
major, n (%)	13 (68.4)	11 (61.1)	
other, n (%)	5 (26.3)	6 (33.3)	
Time between fracture and start study (days)^d^	97.8 ± 34.9	121 ± 41.0	0.104
Use of Calcium before baseline, n (%)^b^	16 (84.2)	15 (83.3)	1.000
Use of vitamin D before baseline (local and systemic), n (%)^b^	17 (89.5)	16 (88.9)	1.000
Use glucocorticosteroids, n (%)^b^	2 (10.5)	2 (11.1)	1.000
Calcium (mmol/l)^a^	2.4 ± 0.1	2.5 ± 0.1	0.713
Albumin (g/l)^a^	42.1 ± 2.3	41.8 ± 2.3	0.722
Vitamin 25(OH)D_3_ (nmol/l)^a^	52.2 ± 13.6	66.9 ± 16.4	**0.005**
PTH (pmol/l)^a^, ^e^	6.2 ± 2.7	5.1 ± 2.9	0.255
Potassium (mmol/l)^a^	4.4 ± 0.3	4.5 ± 0.3	0.378
Sodium (mmol/l)^a^	141.0 ± 1.3	141.0 ± 1.8	0.218
Creatinine (µmol/l)^a^	68.8 ± 7.4	69.1 ± 8.6	0.920
Phosphate (mmol/l)^a^	1.2 ± 0.2	1.2 ± 0.1	0.797
Hemoglobin (mmol/l)^a^	8.7 ± 0.5	8.6 ± 0.7	0.991
Serum CTX (ng/ml)^a^, ^e^	396 ± 150	379 ± 130	0.709
Serum PINP (ng/ml)^a^, ^e^	60.2 ± 12.0	61.6 ± 25.6	0.833

^a^ Independent-samples t-test; ^b^ Fisher’s exact test; ^c^ Chi-square test; and ^d^ Mann-Whitney U-test

e PTH: Parathyroid hormone; CTX: carboxy terminal telopeptide; and PINP: procollagen type I N-terminal propeptide

Of the 37 patients, 36 completed the first 12 weeks (Figure 1). One woman dropped out using branded alendronate because of stomach pain, nausea, reflux and pyrosis. 

Pill counts showed good adherence to the study medication. There was no significant difference in mean pill counts between generic and branded alendronate at week 4 or week 12. For generic and branded alendronate at week 4, the pill count was 98.9%, and 100% and 98.5% at week 12, respectively (p > 0.05). Adherence was also evaluated by SEAMS and BMQ. Again, no significant differences were found at week 4 and 12 between treatments, indicating similar medication adherence (p > 0.05). 

### Tolerance

At baseline, week 4 and 12, 2 (5.4%), 12 (16.2%) and 14 (18.9%) woman did not complete the entire GSRS questionnaire, respectively. Total GSRS scores ranged from 0 to 3.50. The interaction of time*treatment was not significant (p = 0.839). There was no significant differences in tolerance between the two treatment groups at baseline (estimated mean difference (B): 0.11; 95%CI: -0.29 to 0.51), week 4 (B: 0.08; 95%CI: -0.33 to 0.49) and week 12 (B: 0.19; 95%CI: -0.22 to 0.61). 

Subscales of the GSRS were analyzed separately. For the subscale abdominal pain was the interaction of time*treatment not significant (p = 0.254). However, patients using generic alendronate had a significantly higher mean GSRS score at week 4 (B: 0.40; 95%CI: 0.05 to 0.74). At week 12, this mean difference was no longer significant (B: 0.19; 95%CI: -0.17 to 0.54). With regard to the other subscales (diarrhoea, indigestion, constipation and reflux) no significant treatment differences were found (data not shown). 

Self-reported side effects are presented in Table 2. In week 4, 12 patients with generic and 12 patients with branded alendronate did not report any side-effects. This was 14 and 10, respectively, at 12 weeks. The most frequently reported side effects were nausea, constipation and abdominal pain in week 4, and constipation and flatulency/bloating in week 12 (Table 2). For the number of side effects was the interaction of time*treatment not significant (between the treatment groups (p = 0.634). The number of side effects per patient was not significantly different at week 4 (B: 0.13; 95%CI: -0.30 to 0.57) and week 12 (B: -0.16; 95%CI: -0.60 to 0.28). The results for adherence and tolerance did not change when the analysis was performed for the whole cross-over period of 2x12 weeks. There was no significant differences in tolerance between the two treatment groups at week 4 (p = 0.905) or week 12 (p = 0.770) and the score on the subscale of abdominal pain was significantly higher for patients using generic alendronate at week 4 (p = 0.029), but not at week 12 (p = 0.261). The number of side effects was not significantly different at week 4 (p = 0.177) and week 12 ( p = 0.850). Two additional women dropped out after the first 12 weeks, one because of stomach pain, nausea, reflux and pyrosis, and one woman was admitted to the hospital during the study because of an acute coronary syndrome. Both women were using branded alendronate at the time of drop out. No further serious adverse events occurred during the study.

**Table 2:** Self-reported side effects of generic and branded alendronate at week 4 and 12 of the study.

**Side effect**	**Generic alendronate, n = 19 (%)**	**Branded alendronate, n = 18 (%)**	**Total, n = 37 (%)**
***Week 4***			
Reflux	0 (0.0)	1 (5.7)	1 (2.7)
Nausea	2 (10.5)	1 (5.7)	3 (8.1)
Constipation	2 (10.5)	1 (5.7)	3 (8.1)
Flatulency/Bloating	0 (0.0)	1 (5.7)	1 (2.7)
Dysphagia	1 (5.3)	0 (0.0)	1 (2.7)
Diarrhoea	1 (5.3)	0 (0.0)	1 (2.7)
Abdominal pain	2 (10.5)	1 (5.7)	3 (8.1)
Hypertension	1 (5.3)	1 (5.7)	2 (5.4)
Articular pain/spasm	2 (10.5)	0 (0.0)	2 (5.4)
Fatigue	0 (0.0)	1 (5.7)	1 (2.7)
***Week 12***			
Reflux	0 (0.0)	2 (11.1)	2 (5.4)
Nausea	1 (5.3)	0 (0.0)	1 (2.7)
Constipation	1 (5.3)	2 (11.1)	3 (8.1)
Flatulency/Bloating	0 (0.0)	3 (16.7)	3 (8.1)
Abdominal pain	1 (5.3)	0 (0.0)	1 (2.7)
Hypertension	1 (5.3)	0 (0.0)	1 (2.7)
Articular pain/spasm	1 (5.3)	1 (5.7)	2 (5.4)
Fatigue	1 (5.3)	0 (0.0)	1 (2.7)
Pollakisuria	1 (5.3)	0 (0.0)	1 (2.7)
Headache	0 (0.0)	1 (5.7)	1 (2.7)

### Bone turnover markers

**Figure 2:**
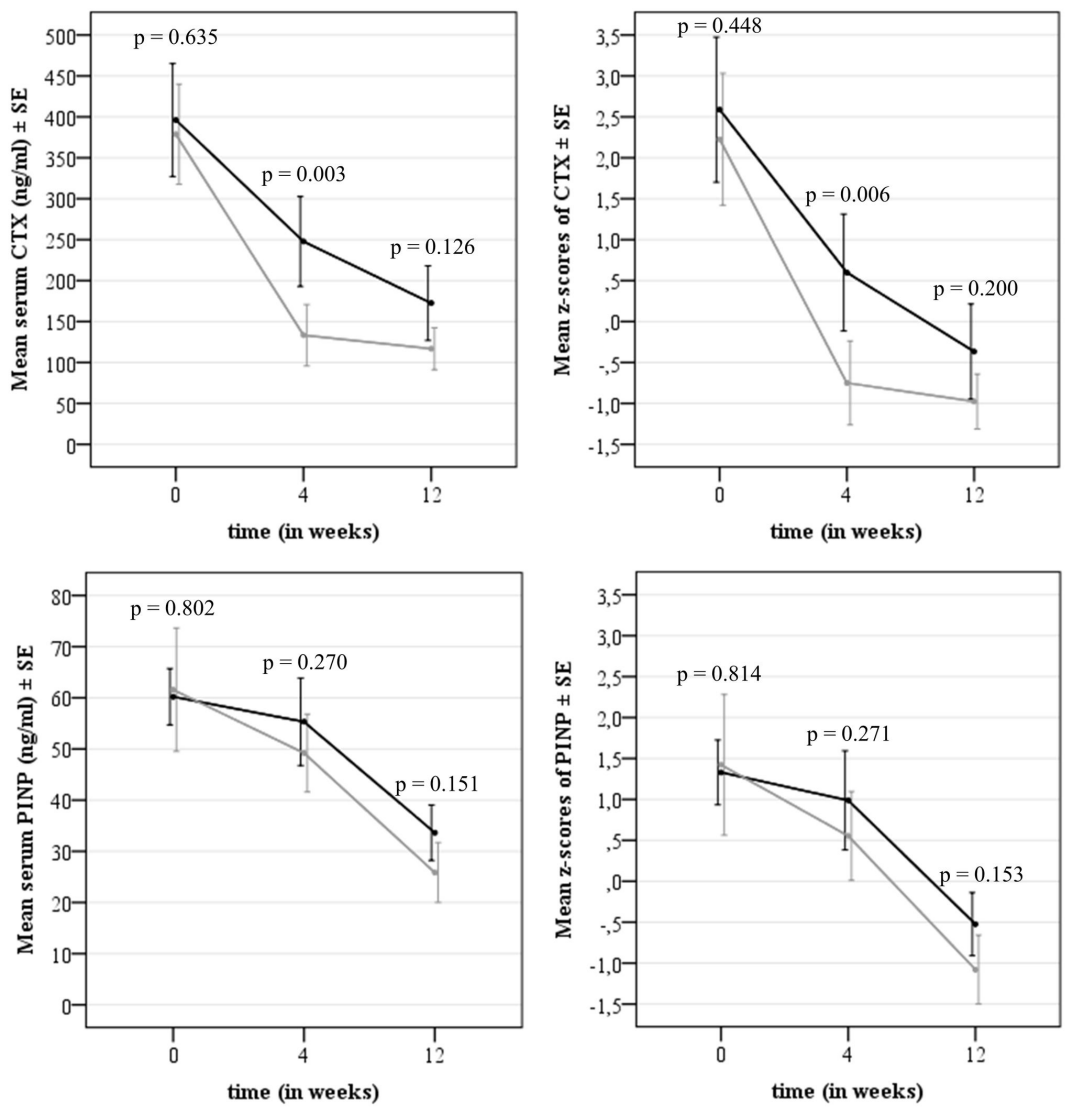
Results of linear mixed models analysis without adjustments for tolerance; estimated means (ng/ml) and standard errors (SE) of CTX (a) and PINP (c) and Z-scores of CTX (b) PINP (d).

The observed unadjusted mean scores and Z-scores of serum CTX and PINP are summarized in Table 3. At baseline, high Z-scores were found in both groups, as expected in patients that sustained a recent fracture. Mean unadjusted CTX and PINP levels decreased significantly over the 12 week follow-up with both generic and branded alendronate (Table 3, p < 0.001). For the mean level of CTX and CTX Z-score there was a significant time*treatment interaction (p = 0.009 and p = 0.046, respectively). At baseline, there was no significant difference between mean CTX level (estimated mean difference (B): 17.4; 95%CI: -55.5 to 90.3). However, the mean CTX level was significantly lower in women treated with branded alendronate at week 4 (B: 114.5; 95%CI: 41.6 to 187.4), but not anymore at week 12 (B: 56.9; 95%CI: -16.4 to 130.3). CTX Z-scores were significantly lower for branded alendronate at week 4 (B: 1.35; 95%CI: 0.40 to 2.30), but not at week 12 (B: 0.62; 95%CI: -0.34 to 1.58). ). For the mean level of PINP and PINP Z-score there was no significant time*treatment interaction (p = 0.202 and p = 0.201, respectively). No significant differences were found at week 4 and 12 for PINP or PINP Z-scores (Table 4, Figure 2). 

**Table 3:** Observed mean (±SD) serum CTX and PINP levels and Z-scores in the patients with generic and branded alendronate.

**Bone marker^a^**	**Generic alendronate (n = 19)**	**Branded alendronate (n = 18)**
***Serum CTX (ng/ml)***		
baseline	396 ± 150	379 ± 130
week 4	248 ± 119	133 ± 79.6
week 12	172 ± 99.2	117 ± 52.7
***Z-scores CTX***		
baseline	2.59 ± 1.93	2.23 ± 1.71
week 4	0.60 ± 1.55	-0.75 ± 1.09
week 12	-0.37 ± 1.27	-0.98 ± 0.69
***Serum PINP (ng/ml)***		
baseline	60.2 ± 12.0	61.6 ± 25.6
week 4	55.3 ± 18.6	49.2 ± 16.1
week 12	33.6 ± 11.8	25.9 ± 12.1
***Z-scores PINP***		
baseline	1.33 ± 0.86	1.42 ± 1.82
week 4	0.99 ± 1.32	0.55 ± 1.15
week 12	-0.52 ± 0.84	-1.08 ± 0.87

a CTX: carboxy terminal telopeptide; PINP: procollagen type I N-terminal propeptide; significant differences between mean unadjusted baseline and week 12 (p < 0.001)

**Table 4:** Estimated mean difference with 95% confidence interval between brackets for serum and z-score CTX and PINP with and without adjustment for tolerance (measured as time-dependent co-variate) at week 4 and 12.

	**Without adjustment for tolerance**		**With adjustment for tolerance**	
	**Week 4**	**Week 12**	**Week 4**	**Week 12**
**CTX (ng/ml)**	114.5	56.9	119.7	55.6
	(41.6 to 187.4)	(-16.4 to 130.3)	(41.8 to 197.6)	(-24.6 to 135.8)
**CTX (z-scores)**	1.35	0.62	1.42	0.56
	(0.40 to 2.30)	(-0.34 to 1.58)	(0.40 to 2.43)	(-0.48 to 1.61)
**PINP (ng/ml)**	6.12	8.06	6.04	6.19
	(-4.86 to 17.1)	(-3.02 to 19.1)	(-5.68 to 17.8)	(-5.94 to 18.3)
**PINP (z-scores)**	0.43	0.57	0.42	0.44
	(-0.35 to 1.22)	(-0.22 to 1.36)	(-0.42 to 1.26)	(-0.42 to 1.30)

After adjustment for tolerance (GSRS, measured at each time point), similar results were found for serum CTX and z-score CTX as well as for serum PINP and z-score PINP (Table 4). 

## Discussion

This study showed that tolerance (GSRS) was similar with generic and branded alendronate in the first 12 weeks of treatment. However, at short-term (4 weeks) the subscale of GSRS regarding abdominal pain was significantly higher with generic compared to branded alendronate. This was no longer significantly different at 12 weeks. The number of self-reported gastrointestinal side effects per patient was not significantly different with generic compared to branded alendronate. The results of other studies suggested that generic alendronate might not be as well tolerated as branded alendronate [20,21]. It was reported that as a result of automatic replacement enforced by the pharmacy, switching from branded to generic alendronate resulted in a significant increase in the frequencies of gastrointestinal side effects [20]. The rate of gastrointestinal events was significantly higher in patients treated with generic alendronate once-weekly than with branded original bisphosphonate treated patients [20]. As in our study, the most frequent observed side effects were stomach pain, gastrointestinal upset, nausea and reflux [20]. In contrast to our study, compliance of patients treated with generic alendronate was significantly lower than compliance of those treated with branded bisphosphonates [21]. However, the follow-up period in this study was much longer (one year). 

As reported recently by Lai et al.[22], the adherence was similar between patients receiving generic or branded alendronate. Comparable adherence was also reported by Landfeldt et al. [23], although others reported reduced persistence with generic alendronate [24]. The finding of higher mean scores for abdominal pain for patients on generic alendronate in 4 weeks compared with branded may be important in patients that switch from branded to generic alendronate because in can be a reason for patients to stop taking the medication. In our controlled-trial setting we did not see a negative effect on adherence but in clinical practice this may be well the case [24]. 

The rapid decrease of bone turnover markers at four weeks achieved with both alendronate formulations, followed by a more gradual decrease at week 12 in patients with elevated bone turnover markers levels because of a recent fracture in our study is in line with the findings in postmenopausal women with osteoporosis treated with alendronate [25,26]. The level of CTX or CTX Z-score was significantly lower at week 4 for branded alendronate, but not anymore at week 12. The difference in effect on bone turnover markers between branded and generic alendronate may be due to the different pharmacokinetic profiles between branded and generic alendronate[14,27]. In vitro-studies revealed important differences in disintegration en dissolution profiles between Fosamax^®^ and nine variations of generic alendronate, that potentially could reduce the efficacy of the generic drugs [14,27]. A slower disintegration profile can lead to accumulation of the semi-disintegrated drug within the oesophagus; thereby enlarge the possibility of contact with ingested food or saliva and reducing the bioavailability of the drug. On the other hand, faster disintegration and dissolution of bisphosphonates could increase the exposure of the agent to oral and oesophageal tissue, resulting in development of mucosal irritation, inflammation and ulceration, which on their part can lead to side effects [28,29]. One study, which compared the disintegration and dissolution of once weekly original branded alendronate (Fosamax^®^) with 26 different generic alendronate copies from Canada, Germany, the Netherlands and UK, showed similar results [27]. The mean disintegration times of the generic alendronate tablets ranged from 14 to 342 seconds, while the mean disintegration times of the branded Fosamax and Actonel ranged from 43 to 78 seconds. Six of twenty-six generic alendronate tablets had very fast disintegration times, up to three times more rapid than the branded Fosamax [27]. 

The results of this study imply potential differences in efficacy, as measured by bone turnover markers, between the investigated generic formulation and branded alendronate in patients with a recent fracture. Whether these differences are clinically relevant and can be extrapolated to patients with osteoporosis but without recent fracture or other formulation of generic alendronate needs to be explored in future studies. However, previously data from the Fracture Intervention Trial revealed that greater changes in bone turnover markers following treatment with antiresorptive agents were associated with greater reduction in fracture risk [30]. The findings of a more rapid and greater decrease in CTX at 4 weeks suggest a different effect on bone turnover markers for the investigated generic compared to the branded alendronate formulation. 

This study has several limitations. First, the study was conducted in only a small number of patients. There was a significantly lower 25(OH)D3 baseline concentration in the group that started with generic alendronate. However, during the study both groups were supplemented with the same vitamin D dose resulting in comparable 25(OH)D3 levels in both groups during treatment, so we expect that the base-line 25(OH)D3 level difference did not influence bone turnover marker results at week 4 and 12. Due to ethical reasons a wash out period was not allowed in this study since all patients needed to be treated with an anti-osteoporosis agent because of their fracture risk profile. As consequence of a carry-over effect on bone turnover markers we could not perform the complete cross-over analysis and had to limit the analyses to the first parallel period of 12 weeks Nevertheless, even with this small study population significant results were found for CTX and for the subscale abdominal pain for tolerance (GSRS). 

In conclusion, bone turnover markers were significantly reduced with branded and generic alendronate in patients with a recent fracture, but overall serum CTX was significantly lower at week 4, but not at week 12, with branded alendronate. Generic alendronate caused significantly more abdominal pain during the first weeks of treatment. Whether this is of clinical relevance cannot be determined based on this study. Based on these findings, generic alendronate may not have the same tolerability and efficacy as branded alendronate in patients with a recent fracture in the first weeks after starting treatment. Further research, preferably in a larger randomized controlled trial with tolerability, bone turnover markers and bone mineral density is needed to assess the magnitude of these potential differences and their clinical implications.

## Supporting Information

Checklist S1 **CONSORT Checklist.**
(DOC)

Protocol S1 **Trial Protocol.**
(PDF)
