# Supplementary material for: Comparing Tolerability and Efficacy of Generic versus Brand Alendronate: A Randomized Clinical Study in Postmenopausal Women with a Recent Fracture
Source: PLoS One. 2013 Oct 21;8(10):e78153. doi: 10.1371/journal.pone.0078153 (PMC3804551; doi:10.1371/journal.pone.0078153)
Supplement: Protocol S1 — Trial Protocol. (PDF) [file pone.0078153.s002.pdf]

A cross-over study to compare efficacy and tolerability of brand versus generic alendronate in postmenopausal women with osteoporosis

Principal Investigator:

Dr. JPW van den Bergh, internist-endocrinoloog

VieCuri MC Noord-Limburg

Supported by:

Stichting wetenschappelijk onderzoek Inwendige Geneeskunde te Noord-limburg

Version 6, November 26, 2009

TABLE OF CONTENTS

Page

STUDY TEAM **4**

PARTICIPATING STUDY SITE **4**

PRÉCIS **5**

1. Study objectives **5**

1.1 Primary Objective 5

1.2 Secondary Objective 6

2. BACKGROUND AND RATIONALE **6**

2.1 Background on Condition, Disease, or Other Primary Study Focus 6

2.2 Study Rationale 6

2.2.1 Generic versus branded alendronate 6

2.2.1.1 Scope 7

2.2.2 Surrogate parameters for evaluation of fracture risk reduction 9

3. STUDY DESIGN **12**

4. SELECTION AND ENROLLMENT OF PARTICIPANTS **13**

4.1 Inclusion Criteria 13

4.2 Exclusion Criteria 13

5. STUDY INTERVENTIONS **14**

5.1 Interventions, Administration, and Duration 14

5.2 Handling of Study Interventions 15

5.3 Assessment of side-effects, adherence and medication use 15

5.4 Evaluation of Bone markers 16

5.5 Evaluation of blood chemistry 16

6. STUDY PROCEDURES **16**

6.1 Schedule of Evaluations 17

6.2 Description of Evaluations 18

7. REMOVAL AND REPLACEMENT OF SUBJECTS 21

7.1 Removal of Subjects 21

7.2 Replacement of Subjects 22

8. SAFETY ASSESSMENTS 22

8.1 Section 10 WMO event 22

8.2 Adverse Events 22

8.3 Serious Adverse Events 23

8.4 Premature termination of the study 23

8.5 Reporting Procedures 24

8.6 Insurance 25

9. STATISTICAL CONSIDERATIONS 25

10. PARTICIPANT RIGHTS AND CONFIDENTIALITY 25

10.1 Institutional Review Board (IRB) Review 25

10.2 Informed Consent Form 25

11. ETHICAL CONSIDERATIONS 26

12. PUBLICATION OF RESEARCH FINDINGS 26

13. REFERENCES 28

STUDY TEAM

VieCuri MC voor Noord-Limburg

Dr. J. van den Bergh, internist-endocrinoloog, prinipal investigator

Mevr. A. van der Velde, studiecoördinator

Mevr. M. Creemers, osteoporoseverpleegkundige

Mevr. A. Kessels, osteoporoseverpleegkundige

Mevr. Drs. N. Drabbe, ziekenhuisapotheker

Dr. M. Janssen, klinisch chemicus

UMC Groningen

Mevr. Dr. E. van der Veer, klinisch biochemicus

PARTICIPATING STUDY SITE

VieCuri Medisch Centrum voor Noord-Limburg

PRÉCIS

Study Title

A cross-over study to compare efficacy and tolerability of brand versus generic alendronate in postmenopausal women with osteoporosis

Objectives

Primary objective: Evaluation of difference in efficacy as measured by bone markers between brand versus generic alendronate in postmenopausal women with osteoporosis

Secondary objective: Evaluation of difference in tolerability between brand versus generic alendronate

Design and Outcomes

A randomized single centre cross-over study to test the tolerability and efficacy of brand compared to generic alendronate in postmenopausal women with established osteoporosis.

Efficacy is evaluated by the assessment the bone resorption marker sCTx. It is hypothesised that there is no difference between brand versus generic alendronate

Tolerability is evaluated by the Gastrointestinal Symptom Rating Scale (GSRS) questionnaire and self-reported side-effects.

Medication use and adherence is evaluated by the Self-efficacy for Appropriate Medication Use (SEAMS) Questionnaire and the Brief Medication Questionnaire (BMQ).

Interventions and Duration

After randomization, patients start with brand alendronate or generic oral alendronate once weekly in a single blinded, open label setting. After the first period of 12 weeks, there is a cross-over to generic and brand oral alendronate once weekly respectively. Evaluation of efficacy, tolerability and adherence is planned at week 4, 12, 16 and 24. After the study period of 24 weeks patients will continue treatment with the alendronate formulation they prefer, according to current guidelines.

Sample Size and Population

30 postmenopausal women with a prior fracture and osteoporosis defined as a DEXA T-score ≤ -2.5 SD at one of the measured locations: lumbar spine, femoral neck or total hip.

1. Study objectives

1.1 Primary Objective

Evaluation of difference in efficacy as measured by bone markers between brand versus generic alendronate in postmenopausal women with osteoporosis

1.2 Secondary Objective

Evaluation of difference in tolerability between brand versus generic alendronate in postmenopausal women with osteoporosis

2. BACKGROUND AND RATIONALE

2.1 Background on Condition, Disease, or Other Primary Study Focus

Osteoporosis is a progressive skeletal pathology characterized by loss of bone mass and quality and development of brittle bones that results in increased risk of fractures. The estimated worldwide number of new osteoporotic fractures for the year 2000 was 9.0 million, of which 1.6 million were at the hip, 1.7 million were at the distal forearm and 1.4 million were clinical vertebral fractures [1]. In the Netherlands the number of osteoporotic fractures is estimated at 80.000 per year (RIVM). Osteoporotic fractures have extensive clinical and economic consequences, and are a major public health concern. The important burden of osteoporotic fractures highlights the need for osteoporosis therapies with established high efficacy. Treatment options for osteoporosis have substantially improved in recent years, appropriate antiresorptive or anabolic medications are available to prevent future fractures. At present, bisphosphonates are the most common therapy for osteoporosis.

Bisphosphonates inhibit osteoclast activity. Two chemically distinct groups of bisphosphonates exist: non-nitrogen-containing bisphosphonates (e.g. Etidronate) and nitrogen- containing bisphosphonates, (e.g. Ibandronate, Alendronate, Zoledronate, and Risedronate).[2, 3] There are two fundamentally distinct components of the mechanism of action of nitrogen-containing bisphosphonates (N-BPs). These are (1) binding to the bone, and (2) binding to and inhibition of a key osteoclast enzyme farnesyl pyrophosphate synthase (FPPS).

Bisphosphonates are today's worldwide leading medication.[4-6] Regarding the oral bisphosphonates, only Risedronate and Alendronate have been proven to reduce vertebral and hip fracture risk in clinical trials. Zoledronate is an intravenous bisphosphonate therapy and has also been proven to reduce vertebral and hip fracture risk in clinical trials. Ácording to the current Dutch guidelines (NHG standaard osteoporose 2005 and CBO richtlijn osteoporose 2002) alendronate and risedronate are the first-choice drugs for treatment of osteoporosis.

2.2 Study Rationale

2.2.1 Generic versus branded alendronate

2.2.1.1 Scope

During the past years an increasing number of generic alendronates were introduced, in numerous countries. For generic drugs the EMEA/CBG guidelines require a bioequivalence study in which the pharmacokinetic profile of the brand formulation (i.e. alendronate) is compared to the reference generic product. Those studies are performed in healthy volunteers. Based on bioequivalence the generic product is expected to have the same clinical efficacy as the brand formulation. Insurances and Health Care Providers prefer physicians to prescribe mainly generic alendronate instead of branded original bisphosphonates like Fosamax® (Alendronate) or Actonel® (Risedronate). Little is known, however, about the provenience and quality of the generic Alendronates. Clinical trials or observational postmarketing studies on bone mineral density and fracture efficacy, adverse events and compliance or persistence with these new "Alendronic acids" initiatives were never performed. This is an important gap of knowledge and means that generic Alendronates are prescribed while it is not known whether efficacy and safety are really the same as was demonstrated for the original branded oral bisphosphonates in clinical or real practice studies. A major concern about the generic alendronates seems to be the reduced medication compliance in clinical practice. An additional issue is related to the poor oral absorption of bisphosphonates in the gastrointestinal tract: typically less than 1% is absorbed. The poor absorption of bisphosphonates is most likely due to their very poor lipophilicity and charge, which prevents transcellular transport across the epithelial barriers. The question arises whether the absorption of generic alendronates, and thus its bioavailability, is altered because of the variation in galenic/tablet properties. This can potentially affect the clinical outcome in terms of anti-fracture efficacy of this medication.

2.2.1.2 Literature overview

In-vitro studies have demonstrated important differences in disintegration en dissolution between Fosamax® and nine variations of alendronic acid.[7, 8] Epstein et al. showed a greater irritant response from a copy Alendronate tablet (Novo-Alendronate 10 mg) in a rabbit injection study and in a dog esophageal study (Alendronate Sodium Tablets, Teva Industries, Petah- Tikva, Israel) compared to the branded innovator product (Alendronate sodium tablets, 10 mg). The differences were attributed to the pharmaceutical preparation, since the active ingredient (Alendronate sodium) and the dose were similar between the copy Alendronate tablets and the branded tablets.[7] The same authors also evaluated the disintegration and dissolution profies of 13 copy Alendronate tablets available. From a safety perspective, the authors concluded that for the rapidly disintegrating formulations there is a chance that disintegration may occur in the mouth and/or the esophagus during swallowing of the tablet. This could increase the duration and extent of oral and esophageal tissue exposed to semi-particulate alendronate and thereby increase the risk of serious mucosal irritation and ulceration [8]. From an efficacy perspective, Epstein et al. also concluded that tablets that disintegrate faster than branded Alendronate sodium tablets (1.4 min) could result in reduced efficacy because the premature disintegration may be associated with semi-particulate Alendronate being retained within the esophagus,

increasing the likelihood of contact with ingested food, saliva, mucus or liquids, thereby reducing the bioavailability or altering the pharmacokinetics. Generic Alendronate tablets with very short disintegration times start to disintegrate very quickly already in the mouth and esophagus. The already low intestinal absorption rates of these generic bisphosphonate drugs could be further decreased and full efficacy in terms of BMD increases not reached.

Two recent studies have shown differences in tablets' properties that might explain different rates of adverse events.[9] An in vitro study compared the disintegration and dissolution of once weekly original branded Risedronate (Actonel®) and original branded Alendronate (Fosamax®) tablets with 26 different Generic Alendronate copies from Canada, Germany, the Netherlands, and UK. The mean disintegration times of the generic alendronate tablets in vitro ranged from 14 to 342 s (5.7 min). The mean disintegration time of the branded product tablets (Actonel® and Fosamax®) ranged only from 43 to 78 s. Six of the 26 companies market alendronic acid tablets having very rapid disintegration times which are similar to those of orally disintegrating tablets (non-bisphosphonates).

Another trial evaluated and compared esophageal transit times and in vivo disintegration of one branded risedronate and two generic formulations of alendronic acid tablets that are commercially available in Canada and the United Kingdom.[10] It was shown that the two generic formulations of alendronic acid tablets had significant slower transit times than compared with the branded risedronate (Actonel®) tablet tested. Also a difference in adhesiveness of generic alendronate compared to Fosamax® has been postulated to contribute to this problem.[11]

This is of importance for patients because delayed esophageal transit or disintegration of oral bisphosphonate tablets before they enter the stomach could cause iatrogenic complications. Different formulations of generic bisphosphonate tablets meeting regulatory requirements may have substantial differences in pharmaceutical attributes from the branded product that may result in different characteristics during esophageal transit.

It has been suggested that there are differences in persistence between different weekly oral bisphosphonaten therapies, particularly a poor persistence for patients started on generic alendronate[4]. In one study a higher incidence in gastro-intestinal side effects was reported in patients treated with generic alendronate as compared to those treated with original once weekly bisphosphonates [12]. In a recently published paper the persistence of patients treated with generic alendronate was significantly lower as compared to those with with branded alendronate. The total numbers of patients reporting gastrointestinal adverse events with generic alendronate was more than twice the number treated with Fosamax®. There was a significantly lower increase of lumbar spine and total hip BMD with generic alendronate compared to Fosamax®. The reasons for the 40–50% lower BMD increase rates when using the generic compound is not known yet. At least in part the lower efficacy can be explained by a significantly lower degree of persistence with generic alendronate, which could be related to a higher incidence of gastrointestinal side events. Other reasons could

be the lower bioavailability or potency of generic alendronate.[13]

Based on these results it is postulated that rapid disintegration may enhance esophageal drug exposure leading to more gastro-intestinal side effects with a possible lower degree of persistence. On the other hand, longer disintegration times may result in altered, possibly delayed or impaired absorption of alendronic acid. Since absorption is less than 1% in branded alendronate there is concern about efficacy of generic formulations.

2.2.2 Surrogate parameters for evaluation of fracture risk reduction

2.2.2.1 BMD

In contrast to other generic medications like statins, anti-diabetic or anti-hypertensive drugs (where you can measure surrogate parameters for evaluation of cardiovascular risk in daily practice such as cholesterol, glucose, HbA1C, blood pressure) it is difficult to evaluate the anti-fracture efficacy of anti-osteoporotic drugs.

BMD as measured by DEXA is a tool developed by the WHO for evaluation of fracture risk and diagnosis of osteoporosis. With each SD decrease in BMD, the fracture risk doubles. The diagnosis osteoporosis is based on a BMD T-score ≤ -2.5SD of the peak bone mass of healthy women between 20 and 30 years. A T-score between -1 and -2,5 is classified as osteopenia en a T-score ≥ -1 as normal. However, this technique is not developed and not useful for evaluation of fracture risk (reduction) during therapy, since the outcome (either increase or decrease of BMD) does not reflect anti-fracture efficacy on an individual basis. Furthermore, it takes at least 2 years to measure a true difference between 2 longitudinally BMD measurements due to the small gain during therapy (3-4% in two years for most drugs) in relation to the precision error of the technique (1-2%). In the Dutch guidelines (CBO 2002 and NHG 2005) it is recommended not to perform a BMD measurement for evaluation of therapy at all. It is advised to use this technique only for the diagnosis of osteoporosis and deciding whether to start treatment or not.

2.2.2.2 Bone turnover markers

Current biological markers of bone turnover have proven useful in improving fracture risk assessment and monitoring treatment efficacy in postmenopausal osteoporosis.[3, 14, 15] Bone turnover markers mainly reflect bone formation by osteoblasts and bone resorption by osteoclasts.

Osteoblasts synthesize collagen, bone-specific alkaline phosphatase (BALP) and osteocalcin. These parameters are therefore markers of bone formation. During synthesis of collagen the propeptides serum C-propeptide of type I collagen (PICP and PINP) are cleaved. These peptides can be measured in serum as a marker for collagen synthesis. During maturation of

osteoblasts in the skeleton, BALP is expressed. In serum the concentration of alkaline phosphatase is predominantly determined by liver- and bone isoenzyme fractions of alkaline phosphatase. Recent new techniques allow for measuring the bone-specific-isoenzyme (BALP) seperately. Osteocalcin, is a small proteine synthesized by osteoblasts during the late phase of the bone formation process. It contains three gamma-carboxyglutamine groups which enable a high affinity for calcium.

Bone parameters that reflect collagen degredation are the “collagen crosslinks” pyridinoline (PYD) en deoxypyridinoline(DPD) and the crosslinks with amino- and carboxyterminal telopeptides (NTx en CTx). These bone-markers are commercially available as immunoassay. Because of the natural biological variation within these parameters (diurnal rythm), collection time is standardised at morning time. Furthermore the results have to be compared to a gender and age specific reference group preferably expressed in Z (or T) - scores. It is now known that high bone turnover is related to enhanced bone-loss in postmenopausal women [14] and to increased fracture risk.[14, 15] It also has been shown that a decrease in bone-resorption markers predicts fracture-risk reduction in patients treated with anti-resoptive drugs.[16, 17] These aspects can be assessed by measuring bone-resorption markers like NTx and CTx (figure 1).

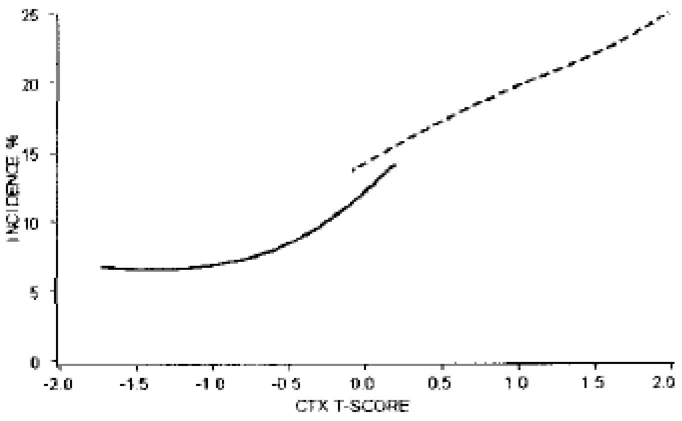

Figure 1: relationship between bone resorption markers expressed as a T-score and the 0- to 3-year incidence of new vertebral fractures. The placebo group is represented by the broken line and the risedronate 5 mg group by the solid line.[16]

In contrast to BMD measurements, the alteration (decrease) in resorption markers occurs in the early phase during treatment. In fact, during the first 3 months of treatment with anti-resorptive medication such as bisphosphonates a decrease of 40-60% of bone turnover markers has been demonstrated (figure 2).[16]

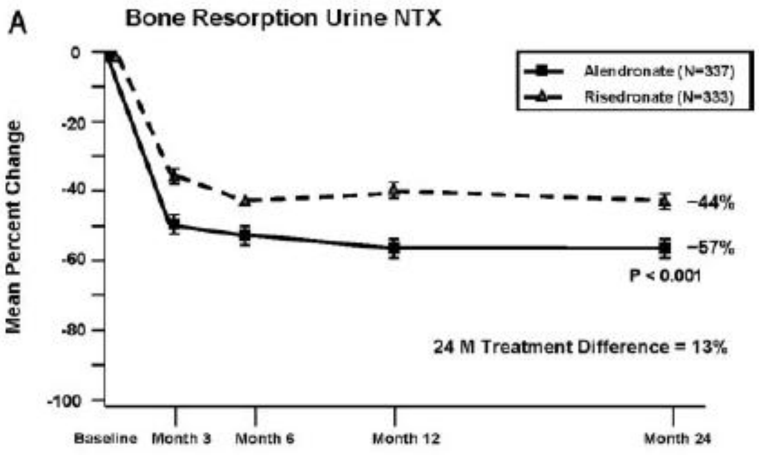

Figure 2: Changes in biochemical markers expressed as mean percentage change from baseline [16]

The predicitive value of a decreased urine NTx for fracture risk reduction was 52%, significantly better than the predicitive value of 14% with femoral neck BMD.[3, 16]

At present, there can be no assurance that the Generic Alendronate tablets are quivalent to the branded product in terms of efficacy and tolerability. In order to gather more information with regard to efficacy and tolerability of branded compared to generic alendronate we designed this study in postmenopausal women with osteoporosis .

3. STUDY DESIGN

- **Type/design of trial:** cross-over design
- **Primary outcome:**

  Comparison of efficacy as measured bone -markers (serum CTx) between generic and brand alendronate formulations

  With regard to the primary outcome we hypothesise that there is no difference between brand versus generic alendronate
- **Secondary outcome:**

  Comparison of tolerability between brand and generic alendronate formulations
- **Study population and sample size:**

  30 postmenopausal women with a prior fracture and osteoporosis, as diagnosed by DEXA (dual X-ray energy), with or without a previous fracture
- **Study location:**

  out -patient, single-centre, VieCuri MC voor Noord-Limburg
- **Duration of screening period and follow-up:**

  The screening period will be a maximum of 2 weeks

  Intervention period: 24 weeks; i.e. 2 times (before and after cross-over) 12 weeks
- **Description of intervention and administration:**

  After randomization, single blinded, open label administration of alendronate

  15 patients start with generic alendronate and 15 patients with brand oral alendronate once weekly

  After the first period of 12 weeks, cross-over to branded and generic oral alendronate once weekly respectively

Evaluation of side-effects, adherence and medication use (by questionnaires) and efficacy (by bone-markers) at day 1, week 4, 12, 16 and 24

After the study period of 24 weeks patients will continue treatment with the alendronate formulation they prefer, according to current practice.

4. SELECTION AND ENROLLMENT OF PARTICIPANTS

Patients will be selected at the out-patient fracture and osteoporosis clinic of the VieCuri MC Noord-Limburg. In this setting all patients are evaluated for possible metabolic bone disease because of a recent fracture by a questionnaire, DEXA and laboratory tests. Based on the results of these diagnostic tests decisions for further evaluation or treatment are made. Therapeutic intervention decisions are made according to the Dutch guidelines (CBO richtlijn osteoporose 2002 en NHG richtlijn osteoporose 2005) and patients are offered or advised to be treated with an oral bisphosphonaten in case of established osteoporosis. Patients who meet these treatment criteria will be asked by their physician (internist working at the VieCuri MC Noord-Limburg) to participate in this study. The study starts after informed consent has been signed by both patient and the principle investigator (Dr. van den Bergh) with a screening on visit -1 .

4.1 Inclusion Criteria

- Ambulatory postmenopausal women, aged 50 years or older

  (postmenopausal will be defined as no vaginal bleeding or spotting for at least 12 months)

  Diagnosed with osteoporosis as defined by a DEXA measurement with a T-score of ≤ -2.5 SD at one of the measured locations: lumbar spine or femoral neck or total hip

- No treatment for osteoporosis in the 12 months before inclusion
- No fracture within the last 12 weeks
- Ability to understand study procedures and to comply with them for the entire length of the study.

4.2 Exclusion Criteria

- Patients with prior treatment of osteoporosis during the previous 12 months (bisphosphonates, testosterone, hormonereplacement therapy (HRT), selective estrogen receptor modulators (SERMs), or calcitonin)

- Vertebral or hip fracture within the last 6 months
- Patients who were previously intolerant of bisphosphonates
- Patients with disorders of esophageal motility or in whom oral bisphosphonates are contraindicated

  A history of upper GI tract disorder other than esophageal motility disorder is not a reason for exclusion.

  Note: prior or current use of nonsteroidal anti-inflammatory drugs (NSAIDs), proton pump inhibitors (PPIs), histamine (H2) receptor antagonists, aspirin, or glucocorticoids is not a reason for exclusion
- Participation of patients in a clinical trail
- Secondary osteoporosis: primary hyperparathyroidism, renal disease (MDRD < 30 ml/min), untreated primary hyper or hypothyroidism
- Not able to follow the drug and dose instructions
- Known malignancy within the last 5 years
- Alcohol use: > 4 units per day
- Inability or unwillingness of individual to give written informed consent

5. STUDY INTERVENTIONS

Before subjects may be entered into the study, a copy of the site's written independent ethics committee/institutional review board (IEC/IRB) approval of the protocol, informed consent forms is required. All subjects must personally sign and date the consent form before enrollment.

5.1 Interventions, Administration, and Duration

All patients will be treated in an out-patient setting with a commercially available EMEA approved formulation of alendronate for a total of 24 weeks; the brand formulation Fosamax® (manufactured by MSD) 70 mg for 12 weeks followed by a generic formulation (aledronic acid) 70 mg for 12 weeks ore vice versa in a cross-over setting. The medication will be administered by the pharmacist at the polytheek of VieCuri MC Noord-Limburg.

Both alendronate formulations must be taken once a week on the same weekday in the fasting

state with water at least 30 minutes before consuming food or beverages in a supine position.

The 70 mg dosage once a week of brand and generic alendronate is the current standard and registered dosage for the treatment of osteoporosis. During the study there will be no modification of the dose.

Side effects**:** The most common side effect of alendronate is stomach pain. It also may cause nausea, vomiting, bloating, constipation, diarrhea, gas, black stool (due to intestinal bleeding), change in taste perception, and muscle or joint pain. Alendronate may irritate the esophagus, causing ulcers and bleeding. This occurs more often when patients do not drink enough water with alendronate or don't wait 30 minutes before lying down.

A standard amount of 1000 mg Calcium and 800 U vitamin D will be added to the treatment with alendronate in all patients as Calci-chew D3 tablets once a day taken with the evening meal.

5.2 **Handling of Study Interventions**

The study drugs will be delivered at the pharmacy 'polytheek' of the VieCui MC voor Noord-Limburg.

Randomization to generic versus brand alendronate at the start of the study will be performed by the site pharmacist by selecting previously prepared closed envelopes.

At delivery patients are explicitly instructed how to take the study medication. The amount of tablets exceeds the amount needed for the study period and patients are instructed to return the study medication at week 4, 12, 16 and 24 visit. At week 4, 12, 16 and 24 the number of returned tablets will be counted.

Calci-chew D3 is also delivered by the pharmacist at the polytheek.

5.3 **Assessment of side-effects, adherence and medication use**

5.3.1 Side effects

Gastrointestinal side effects are scored at base-line, week 4, 12, 16 and 24 by the Gastrointestinal Symptom Rating Scale (GSRS) questionnaire. The GSRS is a brief, fairly comprehensive assessment of common gastrointestinal symptoms based on 15 questions with regard to the previous 4 weeks before assessment. The GSRS has good reliability and construct validity.[18] For detailed information see appendix 1.

Furthermore self reported side-effect will be monitored at baseline, week 4, 12, 16 and 24.

5.3.2 Adherence and medication use

For this study adherence is defined as taken at least 80% of alendronate in both treatment periods of 12 weeks and is evaluated by pill counts. The amount of Caci-chew D3 tablets taken will be counted but is not incorporated in the assessment of adherence.

Medication use and adherence are evaluated by the Self-efficacy for Appropriate Medication Use (SEAMS) Questionnaire and the Brief Medication Questionnaire (BMQ) respectively.[19, 20]
The SEAMS is a reliable and valid instrument based on 13 questions that may provide a valuable assessment of medication self-efficacy in chronic disease management, and appears also appropriate for use in patients with low literacy skills.[19] This questionnaire will be assessed at week 4, 12, 16 and 24For detailed information see appendix 2.
The BMQ appears more sensitive than other existing tools and may be useful in identifying and diagnosing adherence problems.[19] It will be assessed at visit week 4, 12, 16, 24. For detailed information see appendix 3.

5.4 **Evaluation of Bone markers**

The serum sample for CTX (sCTx bij Roche Elecsys), will be obtained at ay 1, week 4, 12, 16 and 24. SCTx should be collected before 10:00 am and subjects should fast overnight before blood sample collection. A second urine morning sample will be collected at the same time. The samples have to be transported on ice and must be frozen within 1 hour. The results will be expressed in absolute values and in Z-scores.

5.5 **Evaluation of blood chemistry**

Serum calcium, phosphate, albumin, 25 OH vitamin D, PTH, Sodium, potassium, kreatinine and Hb will be collected together with sCTx at the same venous puncture (15 ml at each visit 1,2,3,4 and 5).

6. STUDY PROCEDURES

See schedule 6.1

6.1 **Schedule of Evaluations**

Assessment	Screening: Visit-1 (Day-14 to Day -1)	Baseline, Enrollment, Randomization: Visit 1 (Day 1)	Treatment Visit 2 (W4)	Treatment Visit 3 (W12)	Treatment Visit 4 (W16)	Final Visit Visit 5 (W24)
Informed Consent Form	X					
Demographics	X					X
Medical History	X					
Inclusion/Exclusion Criteria	X	X				
Enrollment/Randomization		X				
Concomitant Medications	X	X	X	X	X	X
General Physical Examination	X	X				X
Vital signs	X	X		X		X
Blood Chemistry		X		X		X
Bone markers		X	X	X	X	X
Urine Analysis		X	X	X	X	X
Treatment Administration Form		X	X	X	X	X
GSRS Questionnaire		X	X	X	X	X
SEAMS Questionnaire			X	X	X	X
BMQ Questionnaire			X	X	X	X
Adverse Events		X	X	X	X	X

6.2 **Description of Evaluations**

6.2.1 **Study Visit Definitions**

- The screening date is defined as the date the informed consent is signed.
- Enrolment date is defined as the date of randomization.
- Day 1 is defined as the day that brand or generic alendronate is dispensed to the subject.
- A study month is defined as a calendar month.
- Randomization must occur within 14 days of the screening and Day 1 must be completed within 72 hours of randomization.
- All on-study (post Day 1) visits should be scheduled from the initial Day 1 visit calendar date. For example, if a subject's Day 1 visit is January 15, then their Month-6 visit should be targeted to occur on July 15.
- If a subject's visit is delayed, their subsequent visit dates should not be shifted, but always calculated based on the Day 1 visit date.

6.2.2 **Study Visit Windows**

All tests and procedures scheduled for Day 1 must occur within 72 hours of the randomization date.

All tests and procedures scheduled for the Week 4, 12, 16 and Week 24 visits must be performed within ± 7 days of the scheduled visit date.

Any missed visits, tests not done and examinations not conducted must be reported as such.

Study procedures for a specific visit may be completed on multiple days as long as all of the procedures are completed within the visit window.

6.2.3 **Study visit description**

Screening

Screening and Day-1 visit cannot occur on the same day.

The following assessments will be performed at screening:

- Sign Informed Consent Form

After signing the informed consent form:

- Key baseline risk characteristics
- Medical and medication history
- Vital signs (including blood pressure and pulse)
- Physical examination
- Determine eligibility

Day 1 Visit (within 72 hours of randomization date)

The following assessments will be performed at Day 1 (all Day-1 procedures must be completed prior to administration of brand or generic alendronate):

- Randomize the subject and obtain treatment assignment
- Vital signs (including blood pressure and pulse)
- Physical Examination (including height and weight)
- Laboratory samples
- Concomitant medication recording
- Adverse event collection
- Administration of GSRS
- Brand or generic alendronate dispensed to subject

Week 4 visit (± 7 days)

- Vital signs (including blood pressure and pulse)
- Laboratory samples
- Concomitant medication recording
- Administration of GSRS, SEAMS and BMQ
- Adverse event collection

Week 12 visit (± 7 days)

- Vital signs (including blood pressure and pulse)
- Physical Examination (including height and weight)
- Administration of GSRS, SEAMS and BMQ
- Laboratory samples
- Concomitant medication recording
- Adverse event collection
- Study drug accountability
- Brand or generic alendronate dispensed to subject (cross-over)

Week 16 visit (± 7 days)

- Vital signs (including blood pressure and pulse)
- Laboratory samples
- Concomitant medication recording
- Administration of GSRS, SEAMS and BMQ
- Adverse event collection

Week 24 FINAL visit (± 7 days)

- Vital signs (including blood pressure and pulse)

- Physical Examination (including height and weight)
- Administration of GSRS, SEAMS and BMQ
- Laboratory samples
- Concomitant medication recording
- Adverse event collection
- Study drug accountability

6.2.4 Impact and duration of study visits on participating subjects

There will be a total number of 6 study visits during a total period of 26 weeks. Each visit takes approximately 30 minutes, so the total study visit time per subject will be 180 minutes. During the study there will be 3 general physical examinations, 5 blood collections, each 15ml (total amount 75 ml, for bone markers and chemistry) and 5 urine collections. Participants have to complete a total number of 13 questionnaires throughout the study.

7. REMOVAL AND REPLACEMENT OF SUBJECTS

7.1 Removal of Subjects

Subjects have the right to withdraw fully or partially from the study at any time and for any reason without prejudice to his or her future medical care by the physician or at the institution.

Withdrawal of full consent for a study means that the subject does not wish to receive further investigational treatment and does not wish to or is unable to continue further study participation. Any subject may withdraw full consent to participate in the study at any time during the study. The investigator will discuss with the subject the most appropriate way to withdraw to ensure the subject's health.

Withdrawal of partial consent means that the subject does not wish to take the investigational product any longer but is still willing to collaborate in providing further data by continuing on study (eg, participate in all subsequent study visits or procedures). Subjects may decline to continue receiving the investigational product at any time during the study. These subjects, as well as those who have stopped receiving the investigational product for other reasons (eg, investigator or sponsor concern) will be asked to participate in one, last visit for completion of the data.

Reasons for removal from the investigational product or study might include:

- administrative decision by the investigator

- ineligibility
- significant protocol deviation
- patient noncompliance
- adverse event
- withdrawal of consent

Should a subject (or a legally acceptable representative) request or decide to withdraw from the study, all efforts will be made to complete and report the observations as thoroughly as possible up to the date of withdrawal. If withdrawn consent is recorded as the reason for either ending investigational product or ending study an attempt should be made to determine the possible underlying reason for withdrawing consent, if applicable, and to record that as the primary reason. All information should be reported.

In addition, if a subject experiences an osteoporosis-related fracture (eg, crush vertebral fracture, hip fracture), the investigator should evaluate the situation and discuss alternative treatment options with the subject.

7.2 Replacement of Subjects

Enrolled subjects who have been removed from this study will not be replaced.

8. SAFETY ASSESSMENTS

8.1 Section 10 WMO event

In accordance to section 10, subsection 1, of the WMO, the investigator will inform the subjects and the reviewing accredited METC if anything occurs, on the basis of which it appears that the disadvantages of participation may be significantly greater than was foreseen in the research proposal. The study will be suspended pending further review by the accredited METC, except insofar as suspension would jeopardise the subjects' health. The investigator will take care that all subjects are kept informed.

8.2 Adverse Events

An adverse event is defined in the International Conference on Harmonisation (ICH) Guideline for Good Clinical Practice as "any untoward medical occurrence in a patient or clinical

investigation subject administered a pharmaceutical product and that does not necessarily have a causal relationship with this treatment.” (ICH E6:1.2)

This definition of adverse events also includes worsening of a pre-existing medical condition. Worsening indicates that the pre-existing medical condition (eg, cancer, diabetes, migraine headaches, gout) has increased in severity, frequency, or duration of the condition or an association with significantly worse outcomes.

The occurrence of a fracture will be assessed at visit week 4, 12, 16 and 24 and will be reported as adverse event.

Interventions for pretreatment conditions (eg, elective cosmetic surgery) or medical procedures that were planned before study enrollment are not considered adverse events

The investigator is responsible for reviewing laboratory test results and determining whether an abnormal value in an individual study subject represents a change from values before the study. In general, abnormal laboratory findings without clinical significance (based on the investigator's judgment) should not be recorded as adverse events; however, laboratory value changes requiring therapy or adjustment in prior therapy are considered adverse events.

8.3 **Serious Adverse Events**

A serious adverse event (SAE) is defined as an adverse event that

- is fatal
- is life threatening (places the subject at immediate risk of death)
- requires in-patient hospitalization or prolongation of existing hospitalization
- results in persistent or significant disability/incapacity
- is a congenital anomaly/birth defect
- other significant medical hazard

A hospitalization meeting the regulatory definition for “serious” is any inpatient hospital admission that includes a minimum of an overnight stay in a health care facility. Any adverse event that does not meet one of the definitions of serious (eg, emergency room visit, outpatient surgery, or requires urgent investigation) may be considered by the investigator to meet the “other significant medical hazard” criterion for classification as a serious adverseevent. Examples include allergic bronchospasm, convulsions, and blood dyscrasias.

8.4 **Premature termination of the study**

In addition of paragraph 8.1 the study will be terminated when (serious) adverse event or other disadvantages occur more often than foreseen or currently known during treatment with either one of the study drugs.

8.5 Reporting Procedures

The investigator is responsible for ensuring that all adverse events (as defined in 8.1 and 8.2) observed by the investigator or reported by subjects are collected and recorded in the subjects' medical records. These adverse events will include the following:

The following adverse event attributes must be assigned by the investigator: adverse event diagnosis or syndrome(s) (if known, signs or symptoms if not known); event description (with detail appropriate to the event); dates of onset and resolution; severity; assessment of relatedness to investigational product and action taken.

If applicable, the relationship of the adverse event to the investigational product will be assessed by means of the question: "Is there a reasonable possibility that the event may have been caused by the investigational product?" The investigator should respond to this question with either Yes or No.

The severity grading scale used in this study is described in Appendix B.

Medically significant adverse events considered related to the investigational product by the investigator will be followed until resolved or considered stable.

It will be left to the investigator's clinical judgment to determine whether an adverse event is related and of sufficient severity to require the subject's removal from treatment or from the study. A subject may also voluntarily withdraw from treatment due to what he or she perceives as an intolerable adverse event. If either of these situations arises, the subject should be strongly encouraged to undergo an end-of-study assessment and be under medical supervision until symptoms cease or the condition becomes stable.

Procedures with regard to SUSARS (Suspected unexpected serious adverse reactions):
The investigator will report expedited the following SUSARs to the METC (Maastricht UMC) and CEM (Viecuri Venlo):

- SUSARs that have arisen in the clinical trial that was assessed by the METC and CEM;
- The remaining SUSARs are recorded in an overview list (line-listing) that will be submitted once every half year to the METC. This line-listing provides an overview of all SUSARs from the study medicine, accompanied by a brief report highlighting the main points of concern.

The expedited reporting will occur not later than 15 days after the investigator has first knowledge of the adverse reactions. For fatal or life threatening cases the term will be maximal 7 days for a preliminary report with another 8 days for completion of the report.

The Medical research involving human subjects Act is applicable for (subjects participating in) this study.

In addition to the reporting of SUSARs, the investigator will submit, once a year throughout the clinical trial, a continuity report to the METC (appendix 9).
After completion of the study the termination form will be completed and reported to the METC within 90 days (appendix 10).

8.6 Insurance

The sponsor/investigator has a liability insurance which is in accordance with article 7, subsection 6 of the WMO.
The sponsor (also) has an insurance which is in accordance with the legal requirements in the Netherlands (Article 7 WMO and the Measure regarding Compulsory Insurance for Clinical Research in Humans of 23th June 2003). This insurance provides cover for damage to research subjects through injury or death caused by the study. (appendix 7)

9. STATISTICAL CONSIDERATIONS

The hypothesis tested in this trial is a non-inferiority in efficacy based on the outcome parameter: serum CTx resorption bone marker at visit 3 and 5 (week 12 and week 24) of both treatment groups.

De CTx outcome will be expressed in standardized Z-scores. Z-scores can be used to compare a measurement to a reference value. The Z-score is the number of standard deviations away from the average value of the age and gender specific reference group (i.e. women of the same age).[16] A difference < 0.5SD (in terms of serum Ctx Z-scores) between brand and generic alendronate is considered not to be clinically relevant.

Sample size is calculated ($N = (Zalpha + Zbeta)^{2} * Sd^{2} / d^{2}$; paired observations) based on α = 0.05 (95% confidence interval), β = 0.1 (power 90%), with a standard deviation of sCTx measurements of 0.5 SD (S) and d as the difference between the mean of both treatment groups. Considering a serum CTx Z-score difference of < 0.5 SD between both groups as not clinically relevant, a sample size of 30 patients is needed to test the non-inferiority hypothesis.

10. PARTICIPANT RIGHTS AND CONFIDENTIALITY

10.1 Institutional Review Board (IRB) Review

This protocol and the informed consent document - Appendix 4 - and any subsequent modifications will be reviewed and approved by the ethics committee responsible for oversight of the study.

10.2 Informed Consent Form

See appendix 4

11. ETHICAL CONSIDERATIONS

All subjects participating in this study will be informed in writing and verbally about the purpose and procedures of the study by the investigator or research coordinator. All subjects will provided an informed consent form describing this study and providing sufficient information for subjects to make an informed decision about their participation in this study. (See Appendix 4: Subject Informed Consent Form). This consent form will be submitted with the protocol for review and approval by the Ethical Committee aZM/UM.
The formal consent of a subject (by signing the approved consent form) must be obtained before that subject undergoes any study procedure. The consent form must be signed and dated by both the subject and the investigator. The subject will be provided with a signed copy of the informed consent.

Subjects' confidentiality will be ensured by the investigator and will be maintained by the following procedures:

- Subjects will be coded by their initials and a specific study number. This code will be used on all study forms or other documents with regard to this study.

  The individual participants number is based on the year and month at entrance of the study, with the adaptation of number 1 to 30. For example: 091201 (start of participation in study in December 2009, 1st participant) or 100125 (start January 2010, 25th participant)

- Documents that are needed in compliance with Federal regulations/ICH GCP guidelines e.g. signed informed consents, subjects identification list, will be kept at a secure location and in strict confidence by the investigator.

Subjects are free at all time to discontinue participating in the study, without providing any reason for discontinuation.

An independent physician is available for questions of the subjects.

Subjects will be informed about the outcome of the trial.

All data collected will be kept for 15 years after completion of the study.

Participating subjects will be insured by Medirisk VieCuri MC voor Noord-Limburg

12. PUBLICATION OF RESEARCH FINDINGS

The CCMO statement on publication policy will be followed (www.ccmo.nl). See appendix 6.

Both positive and negative results of this trial will be disclosed unreservedly and will be submitted for publication in a peer-reviewed scientific journal. None of the parties concerned has a right of veto and disputes on the interpretation of the results may not lead to unnecessary

delay in publication. For detailed information we refer to the CCMO statement on publication policy.

13. REFERENCES

1. Johnell O, Kanis JA. An estimate of the worldwide prevalence and disability associated with osteoporotic fractures. Osteoporos Int 2006;17(12):1726-1733.

2. Stepan JJ. Prediction of bone loss in postmenopausal women. Osteoporos Int 2000;11 Suppl 6:S45-S54.

3. Garnero P. Markers of bone turnover for the prediction of fracture risk. Osteoporos Int 2000;11 Suppl 6:S55-S65.

4. Bilezikian JP. Efficacy of bisphosphonates in reducing fracture risk in postmenopausal osteoporosis. Am J Med 2009;122(2 Suppl):S14-S21.

5. Gass M, Dawson-Hughes B. Preventing osteoporosis-related fractures: an overview. Am J Med 2006;119(4 Suppl 1):S3-S11.

6. Iwamoto J, Takeda T, Sato Y. Efficacy and safety of alendronate and risedronate for postmenopausal osteoporosis. Curr Med Res Opin 2006;22(5):919-928.

7. Epstein S, Geusens P, Fisher JE, Hill SL, Roy S. Disintegration and esophageal irritation profiles of alendronate formulations: implications for clinical safety and effcacy. J Apple Res 2005;5:253-264.

8. Epstein S, Cryer B, Ragi S et al. Disintegration/dissolution profiles of copies of Fosamax (alendronate). Curr Med Res Opin 2003;19(8):781-789.

9. Marx RE. Uncovering the cause of "phossy jaw" Circa 1858 to 1906: oral and maxillofacial surgery closed case files-case closed. J Oral Maxillofac Surg 2008;66(11):2356-2363.

10. Perkins AC, Blackshaw PE, Hay PD et al. Esophageal transit and in vivo disintegration of branded risedronate sodium tablets and two generic formulations of alendronic acid tablets: a single-center, single-blind, six-period crossover study in healthy female subjects. Clin Ther 2008;30(5):834-844.

11. Shakweh M, Bravo-Osuna I, Ponchel G. Comparative in vitro study of oesophageal adhesiveness of different commercial formulations containing alendronate. Eur J Pharm Sci 2007;31(5):262-270.

12. Papaioannou A, Ioannidis G, Grima D, Adachi JD. Quantification and description of gastrointestinal adverse events (GI AES) in patients switched from branded Fosamax® to generic alendronate. abstract. 2008. ISCD.

13. Ringe JD, Moller G. Differences in persistence, safety and efficacy of generic and original branded once weekly bisphosphonates in patients with postmenopausal osteoporosis: 1-year results of a retrospective patient chart review analysis. Rheumatol Int 2009.

14. Garnero P, Sornay-Rendu E, Chapuy MC, Delmas PD. Increased bone turnover in late postmenopausal women is a major determinant of osteoporosis. J Bone Miner Res 1996;11(3):337-349.

15. Garnero P. Bone markers in osteoporosis. Curr Osteoporos Rep 2009;7(3):84-90.

16. Eastell R, Barton I, Hannon RA, Chines A, Garnero P, Delmas PD. Relationship of early changes in bone resorption to the reduction in fracture risk with risedronate. J Bone Miner Res 2003;18(6):1051-1056.

17. Li Z, Meredith MP, Hoseyni MS. A method to assess the proportion of treatment effect explained by a surrogate endpoint. Stat Med 2001;20(21):3175-3188.

18. Revicki DA, Wood M, Wiklund I, Crawley J. Reliability and validity of the Gastrointestinal Symptom Rating Scale in patients with gastroesophageal reflux disease. Qual Life Res 1998;7(1):75-83.

19. Risser J, Jacobson TA, Kripalani S. Development and psychometric evaluation of the Self-efficacy for Appropriate Medication Use Scale (SEAMS) in low-literacy patients with chronic disease. J Nurs Meas 2007;15(3):203-219.

20. Svarstad BL, Chewning BA, Sleath BL, Claesson C. The Brief Medication Questionnaire: a tool for screening patient adherence and barriers to adherence. Patient Educ Couns 1999;37(2):113-124.

APPENDICES

Appendix 1:	GSRS
Appendix 2:	SEAMS
Appendix 3:	BMQ
Appendix 4:	Informed consent
Appendix 5:	SAE form
Appendix 6:	CCMO statement on publication policy
Appendix 7:	Informatie proefpersonen verzekering, versie augustus 2008
Appendix 8	SUSAR meldformulier, versie juni 2006
Appendix 9	Voortgangsrapportage formulier, versie september 2009
Appendix 10	Formulier melding (voortijdige) beëindiging studie, versie augustus 2009
Appendix 11	Contract Procter and Gamble
